# The safety of at home powdered infant formula preparation: A community science project

**DOI:** 10.1111/mcn.13567

**Published:** 2023-10-04

**Authors:** Aimee Grant, Sara Jones, Vicky Sibson, Rebecca Ellis, Abbie Dolling, Tara McNamara, Jonie Cooper, Susan Dvorak, Sharon Breward, Phyll Buchanan, Emma Yhnell, Amy Brown

**Affiliations:** ^1^ Centre for Lactation, Infant Feeding and Translational Research Swansea University Swansea UK; ^2^ First Steps Nutrition Trust, Studio 3.04 The Food Exchange New Covent Garden Market London London UK; ^3^ Betsi Cadwaladr University Health Board, Ysbyty Gwynedd Bangor Gwynedd UK; ^4^ The Breastfeeding Network Paisley UK; ^5^ School of Biosciences Cardiff University Sir Martin Evans Building Cardiff UK

**Keywords:** breast milk substitutes, child health, food safety, infant feeding, infant formula, PIF, powdered infant formula, public health

## Abstract

Formula fed infants experience gastrointestinal infections at higher rates than breastfed infants, due in part to bacteria in powdered infant formula (PIF) and bacterial contamination of infant feeding equipment. The United Kingdom National Health Service (UK NHS) has adopted the World Health Organization recommendation that water used to reconstitute PIF is ≥70°C to eliminate bacteria. We used community science methods to co‐design an at home experiment and online questionnaire (‘research diary’) to explore the safety of PIF preparation compared to UK NHS guidelines. 200 UK‐based parents of infants aged ≤12 months were recruited; 151 provided data on PIF preparation, and 143 were included in the analysis of water temperatures used to reconstitute PIF. Only 14.9% (*n* = 11) of 74 PIF preparation machines produced a water temperature of ≥70°C compared with 78.3% (*n* = 54) of 69 kettle users (*p* < 0.001). The mean temperature of water dispensed by PIF preparation machines was 9°C lower than kettles (Machine *M* = 65.78°C, Kettle *M* = 75.29°C). Many parents did not always fully follow NHS safer PIF preparation guidance, and parents did not appear to understand the potential risks of PIF bacterial contamination. Parents should be advised that the water dispensed by PIF preparation machines may be below 70°C, and could result in bacteria remaining in infant formula, potentially leading to gastrointestinal infections. PIF labelling should advise that water used to prepare PIF should be ≥70°C and highight the risks of not using sufficiently hot water, per WHO Europe advice. There is an urgent need for stronger consumer protections regarding PIF preparation devices.

## Key points

- Only 14.9% (*n* = 11) of 74 PIF preparation machines produced a water temperature of ≥70°C compared with 78.3% (*n* = 54) of 69 kettle users (*p* < 0.001).
- The mean temperature of water dispensed by PIF preparation machines was 9°C lower than kettles (Machine *M* = 65.78°C, Kettle *M* = 75.29°C).
- Most parents routinely washed and sterilised bottles and teats.
- Many parents did not always fully follow NHS safer PIF preparation guidance, including 21.8% washing their hands half the time or less, and 14.6% regularly pre‐preparing bottles.
- Most parents did not appear to understand the risks of PIF bacterial contamination.

## Graphical abstract

Community scientists were involved in all stages of the finding the formula study.

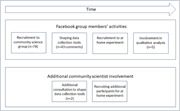

## 1 INTRODUCTION

Almost three quarters of infants in the United Kingdom receive infant formula in the first 6 weeks of life; rising to 88% by 6 months of age, (McAndrew et al., 2012). Most commonly, infant formula comes in the form of powdered infant formula (PIF) which is reconstituted into liquid form by adding water. Gastrointestinal infections in infancy are more prevalent in formula fed infants; at least 3000 hospitalisations may be attributed to formula feeding in the United Kingdom each year (Renfrew et al., 2012). By contrast, breastfed infants experience significantly fewer gastrointestinal infections, due to the antimicrobial and immunity supportive properties of breast milk and the absence of infection risks associated with formula feeding (Victora et al., 2016).

The risk of gastrointestinal infection increases in formula fed infants because of several known mechanisms: the infant formula itself can contain bacteria (Crawley et al., 2022); the feeding equipment may house bacteria if they are not washed or sterilised thoroughly (Redmond et al., 2009) and preparing infant formula with unclean hands can contaminate equipment (Cho et al., 2019). PIF is not, and cannot be made to be, sterile and thus it can contain bacteria including *Salmonella* and *Cronobacter*. There have been many documented outbreaks of bacterial infection related to contaminated PIF (see e.g., Strysko et al., 2020), leading to meningitis, sepsis and death (Centre for Disease Control and Prevention, 2022). For example, a *Cronobacter* outbreak in the United States in 2022 was identified as potentially contributing to the deaths of two children (US Food and Drug Administration, 2022).

To minimise the risk of infection from PIF, The World Health Organization (WHO) advice states that PIF must be mixed with water at a temperature of ≥70°C (158°F) (World Health Organization [WHO], 2007). Accordingly, the UK National Health Service (National Health Service [NHS], 2019) advises boiling fresh tap water in a kettle to prepare PIF at temperatures ≥70°C, and cooling the prepared formula to a drinking temperature, as well as making each feed one at a time as needed (see Box 1). PIF preparation machines are marketed as an alternative to preparing PIF with a kettle, but were not included in NHS guidance (2019) at the time of writing. A survey of over 600 parents in the United Kingdom identified that 56% used a PIF preparation machine some of the time and 45% as their primary PIF preparation method (Brown et al., 2020).

Box 1: **National health service ‘Step‐by‐step guide to preparing a formula feed’ (2019)**

- Step 1: Fill the kettle with at least 1 L of fresh tap water (do not use water that has been boiled before).
- Step 2: Boil the water. Then leave the water to cool for no more than 30 min, so that it remains at a temperature of at least 70 C.
- Step 3: Clean and disinfect the surface you are going to use.
- Step 4: It's important that you wash your hands.
- Step 5: If you are using a cold‐water steriliser, shake off any excess solution from the bottle and the teat, or rinse them with cooled boiled water from the kettle (not tap water).
- Step 6: Stand the bottle on the cleaned, disinfected surface.
- Step 7: Follow the manufacturer's instructions and pour the amount of water you need into the bottle. Double check that the water level is correct. Always put the water in the bottle first, while it is still hot, before adding the powdered formula.
- Step 8: Loosely fill the scoop with formula powder, according to the manufacturer's instructions, then level it using either the flat edge of a clean, dry knife or the leveller provided. Different tins of formula come with different scoops. Make sure you only use the scoop that comes with the formula.
- Step 9: Holding the edge of the teat, put it into the retaining ring, check it is secure, then screw the ring onto the bottle.
- Step 10: Cover the teat with the cap and shake the bottle until the powder is dissolved.
- Step 11: It's important to cool the formula so it's not too hot to drink. Do this by holding the bottle (with the lid on) under cold running water.
- Step 12: Test the temperature of the formula on the inside of your wrist before giving it to your baby. It should be body temperature, which means it should feel warm or cool, but not hot.
- Step 13: If there is any made‐up formula left in the bottle after a feed, throw it away.

PIF preparation machines vary in their design. A common design for PIF preparation machines in the United Kingdom is for them to: dispense a small volume of hot water (a ‘hot shot’) in to the bottle, the bottle should then be promptly removed from the machine to allow PIF to be added by carers manually, the bottle should then be shaken and returned to the machine to be topped up with cool water, and shaken again before feeding. However, the most recent advice from manufacturers recommends adding the PIF before the hot shot, although this is not compliant with NHS (2019) advice. Other PIF machines dispense a bottle of made‐up formula. Concerns have been raised about the safety of both types of formula preparation machine (Food Safety Authority of Ireland, 2021; South Tees Hospitals NHS Foundation Trust, 2022), including the potential for small volumes of water (‘hot shots’) to fail to remain at 70°C for long enough to kill any bacteria in the PIF (Crawley et al., 2022; Norfolk and Norwich University Hospitals NHS Foundation Trust, 2021).

In addition to using sufficiently hot water, those preparing PIF must take steps to minimise contamination of baby feeding equipment, including by washing their hands, disinfecting preparation surfaces and washing and sterilising all feeding equipment. However, research in the USA has shown that parents often do not do this (Labiner‐Wolfe et al., 2008). The NHS (2019) advice on PIF preparation contains 13 steps to reduce the risk of contamination (see Box 1). Although manufacturers of PIF in the United Kingdom are not currently required to include the NHS (2019) advice, or to state the importance of using hot enough water to kill any bacteria present because the PIF is not sterile, on the label. In addition, some PIF manufacturers, recommend using water <70°C. For example, while the NHS (2019) recommend boiling 1 L of water in a kettle and waiting for no more than 30 min, known as the ‘boil to pour time’, to ensure that the water remains >70°C, a boil to pour time of 45 min for 1 L of water is recommended by some manufacturers (e.g., Aptamil, 2022). UK parents report feeling confused and unconfident in relation to preparing PIF (Brown et al., 2020), which may be as a result of a lack of consistent information that is clear and easy to understand.

## 2 METHODS

### 2.1 Aim and objectives

Aim: To explore the feasibility of working with caregivers to collect data in the home relating to the safety of PIF preparation.

Objectives:

1. Engage an online community of community scientists interested in the safety of PIF to co‐design the study and input into data analysis
2. Assess the feasibility of collecting data relating to PIF preparation in the home
3. Gather data on: (a) the range of water temperatures achieved when preparing PIF in the home using a range of PIF preparation methods, and (b) barriers and facilitators to achieving safe temperatures.

This paper reports briefly on objectives 1 and 2, which will be reported on in more detail in a separate paper, but primarily explores the data gathered in relation to objective 3.

### 2.2 Community science approach and research design

Limited attention has been paid to the role that consumers can play in shaping understandings of food safety, despite most food preparation being relatively hidden behaviour, largely confined to the home (Reynolds et al., 2021). Citizen Science is an approach where lay members of the public with relevant lived experience, or an interest in a topic, join research teams and contribute to a range of research‐related activities with appropriate support from academic researchers. We aimed to meet nine of the European Citizen Science Association's *Ten Principles for Citizen Science* (ECSA, 2015) (see Box 2), although we used the term *Community Science* due to the negative racialised connotations of the word ‘citizen’. The one ECSA principle that we were unable to follow related to data sharing. This was due to the potential for harm if the data was provided to manufacturers of breast milk substitutes who undermine breastfeeding by failing to adhere to World Health Organization standards (WHO, 1981).

Box 2: **Summary of ECSA 10 principles for citizen science (2015)**

1. Citizen science projects actively involve citizens in scientific endeavour that generates new knowledge or understanding. Citizens may act as contributors, collaborators or as project leader and have a meaningful role in the project.
2. Citizen science projects have a genuine science outcome.
3. Both the professional scientists and the citizen scientists benefit from taking part.
4. Citizen scientists may, if they wish, participate in multiple stages of the scientific process.
5. Citizen scientists receive feedback from the project.
6. Citizen science is considered a research approach like any other, with limitations and biases that should be considered and controlled for.
7. Citizen science project data and meta‐data are made publicly available and where possible, results are published in an open access format.
8. Citizen scientists are acknowledged in project results and publications.
9. Citizen science programmes are evaluated for their scientific output, data quality, participant experience and wider societal or policy impact.
10. The leaders of citizen science projects take into consideration legal and ethical issues surrounding copyright, intellectual property, data sharing agreements, confidentiality, attribution and the environmental impact of any activities.

**Figure 1:**
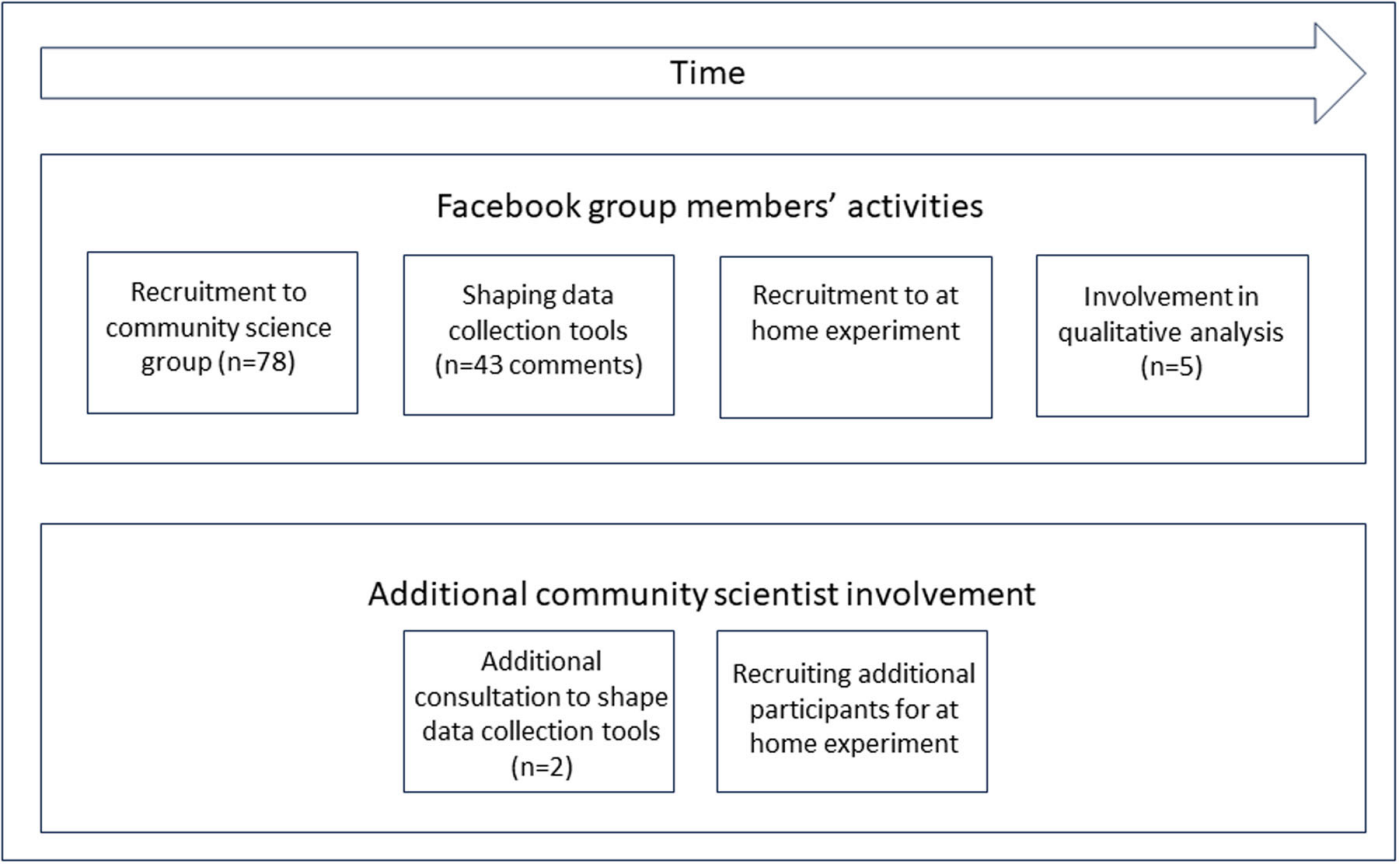
Involvement of community scientists.

We illustrate the points at which community scientists were involved in this study in Figure 1. First, using social media posts on the researchers' and study funders (Food Standards Agency) accounts, we recruited 78 parents of infants aged <12 months who used PIF, to join a closed Facebook group: *Finding the Formula Community Science Project*. Second, we asked members of the closed Facebook group for feedback on our data collection tools (protocol for the at home experiment and research diary) and received 43 comments from group members, and two additional mothers who were not members of the group provided a further in‐depth review of these materials. This resulted in four iterations of the protocol for the at home experiment being developed until only positive feedback was received. Third, members of the Facebook group were invited to complete the ‘at home experiment’ to assess PIF preparation safety, and were also invited to share the study's recruitment materials on social media. Finally, five members of the closed Facebook group contributed to data analysis, and four of them are authors on this paper and the other study outputs. Further outputs detailing this process are in preparation. For clarity, we refer to those involved as community scientists when they were undertaking research design and analysis tasks, but participants during the data collection activities which form this paper's results.

### 2.3 At home experiment and research diary

#### 2.3.1 Eligibility criteria and participant recruitment

The study was advertised in the study's community science Facebook group, and then via the research teams' personal accounts on Facebook, Twitter and Instagram. Potential participants viewed the participant information sheet and an eligibility screen before completing a consent form and providing their postal and email addresses if they were eligible. The eligibility screen, hosted on Qualtrics, determined that participants were:

1. >18 years of age.
2. A parent or caregiver of a baby ≤12‐month‐old using PIF.
3. Living in the United Kingdom.
4. *Not* using a PIF preparation machine which mixed PIF and water inside the machine, producing a finished bottle of formula.

Those who did not meet the eligibility criteria or did not agree to consent were not able to provide their details, so it is not possible to say how many people were excluded at this stage. We excluded those using a PIF preparation machine that *dispensed* infant formula, rather than hot water, for two reasons. First, the formula dispensed would not be comparable to the ‘hot shot’ from other PIF preparation machines or hot water from a kettle. Second, we were concerned that having prepared a bottle of *formula* and inserted a nonsterile thermometer into it, it could be fed to a baby to avoid wasting PIF despite potentially being contaminated.

#### 2.3.2 Materials

Two‐hundred participants were posted a study pack, containing a thermometer and hard copy instruction sheet (see Table 1). Our potential sample size was determined based on the study's budget, as part of assessing the feasibility of this type of community science data collection in this population of participants. The instructions varied between mode of water heating (kettles and similar items V PIF preparation machine) for clarity following review from the community scientists. Those using a kettle or similar to participate were asked to use two bottles (the ‘test bottle’ and the ‘formula bottle’), and to complete the experiment while simultaneously preparing a bottle of PIF (see Supporting Information: Appendix 1). Those using a PIF preparation machine would not be able to prepare two bottles simultaneously, so were instructed to use a ‘test bottle’ at a time when they were not preparing a feed for their baby (see Supporting Information: Appendix 2). Those who reported using both modes of water heating at recruitment were sent both instruction sheets. Both instruction sheets provided space for participants to record the temperature of the water and other relevant details, such as boiling time and pour time for kettle users and volume of water selected for PIF machine users. Participants were also emailed a link to an online survey hosted on Qualtrics, that we called their ‘research diary’, which was to be completed after undertaking the experiment (see Table 1). Feedback on the research diary was provided by community scientists via our study's Facebook group.

**Table 1:** Key resources table.

Resource	Details	Notes
Thermometer	Ashley housewares' digital food thermometers model MT301	Chosen over scientific/medical grade thermometers due to budget constraints. Not calibrated before use.
Instruction sheet: Kettles, baby kettles or instant hot water taps	See Supporting Information: Appendix 1	Instructions for using a ‘test bottle’ and a ‘formula bottle’ simultaneously, using the same volume of water for both How to use the thermometer Space to record experimental results QR code to research diary
Instruction sheet: PIF preparation machines	See Supporting Information: Appendix 2	Instructions for using a ‘test bottle’ to measure the temperature of the ‘hot shot’ only How to use the thermometer Space to record experimental results QR code to research diary
Research diary	Hosted on qualtrics	Demographics Milks fed to baby PIF preparation mode All elements of National Health Service (NHS) (2019) guidance for safer PIF preparation (Box 1) The things that make it easier or more difficult to follow NHS (2019) guidance The experiment: How performed; water temperature Experiences of taking part in the study

#### 2.3.3 Data collection

Recruitment opened in March 2022. All 200 participant packs were posted by May 2022. If participants had not completed their research diary within 2–4 weeks they received up to two reminder emails.

#### 2.3.4 Data analysis

Within a week of research diary completion, Jones, a registered Community Public Health Nurse (‘health visitor’), reviewed research diaries for water temperature data. Participants were followed up by email, thanking them for their participation, sending a £5 shopping voucher and links to information from the NHS and First Steps Nutrition Trust on safer PIF preparation. Participants who recorded lower than advised temperatures (<70°C), were told how to achieve a temperature ≥70°C using a kettle, as per the NHS (2019) advice.

Quantitative data were analysed by Jones using SPSS v.28 (IBM). Descriptive statistics were produced for each question. For the at‐home experiment, responses were divided into two groups for comparison: kettle users and PIF preparation machine users, with the small number of participants using baby kettles and instant hot water taps excluded. Inferential statistics (t‐tests, ANOVA, *χ* ^2^ tests and Pearson's correlations) were performed.

Questions producing open‐ended data were subjected to an inductive thematic analysis (Braun & Clarke, 2022) in collaboration with five community scientists, all mothers (including Dolling, McNamara,  Cooper and Dvorak). Familiarisation and coding, by individual question, was undertaken in NVivo R1 by Ellis. This was discussed and reviewed with Grant, before data extracts were presented by Grant and Ellis to the community scientists through a series of 13 1‐h group analysis sessions, supplemented by asynchronous ways to take part, including via a private online message board only viewable to the five community scientists and email. Analysis meetings were designed following best practice for involving stigmatised community members in analysis, including asking for feedback and making improvements at several points, renumerating community scientists and acknowledging their role in the wider study (Jennings et al., 2018).

#### 2.3.5 Ethical statement

Swansea University's School of Health and Social Care Research Ethics Committee approved this study. Participants freely provided informed consent. All aspects of the study were performed in accordance with the ethical standards set out in the 1964 Declaration of Helsinki.

## 3 RESULTS

### 3.1 Sample characteristics

#### 3.1.1 Participant demographics

Of 200 study packs posted, 151 completed research diaries were received, a response rate of 75.5%. All participants were parents; the majority (*n* = 143; 94.7%) were mothers, with eight (5.3%) fathers. The mean age of parents was 32.87 years (range: 21–43, SD = 4.46). Four parents (2.6%) considered themselves Disabled. Parents were highly educated, with 80.8% (*n* = 122) having at least an undergraduate degree. The mean age of participants' youngest (or only) baby was 7.05 months (range: 1–12, SD = 2.74). Over half (*n* = 88; 58.3%) were first time parents; two (1.3%) people did not answer the question. Among participants with more than one child (*n* = 61; 40.4%), almost all had experience of using PIF with their older child or children (*n* = 56; 91.84%). Four‐fifths of the sample (*n* = 120; 79.5%) reported that they were the person responsible for making their baby's PIF feeds always or most of the time. The question relating to ethnicity was incorrectly entered into the Qualtrics platform used to collect data, and it is therefore not possible to describe the ethnicity of participants.

#### 3.1.2 Types of milk feeds

All parents used PIF, with 84 (55.6%) reporting that all their baby's feeds in the home were PIF, although only 44 (29.1%) exclusively fed their baby PIF. Over three‐quarters of parents (*n* = 116; 76.8%) used standard first infant formula. The next most common type was standard follow‐on formula (*n* = 20; 13.2%). Other preparations of infant formula included ‘ready to feed’ (*n* = 91; 60.3%) and infant formula tablets (*n* = 1; 0.7%). In addition to infant formula, 59 parents (39.1%) also fed their baby breast milk and 12 (7.9%) fed their baby cows' milk.

### 3.2 Preparing PIF in relation to NHS (2019) guide to preparing a formula feed

#### 3.2.1 Water type and method of heating water

Sixty‐seven parents (44.4%) said that they always used fresh tap water, that had not been boiled before. Other common types of water used included, pre‐boiled water that was boiled again (water already in the kettle) (*n* = 26; 17.2%), cooled boiled water (*n* = 37; 24.5%) and filtered water (*n* = 22; 14.6%); some parents selected more than one option. Most parents (*n* = 121; 80.1%) used a regular kettle at least some of the time, with 71 (47%) parents using this method always or most of the time. The majority of kettle users (*n* = 102; 67.5%) said they usually used water within 30 min after boiling, as per NHS advice. Just over half of our sample (*n* = 78; 51.6%) used a PIF preparation machine sometimes. Instant boiling water taps (*n* = 6; 3.9%), baby kettles (*n* = 6; 3.9%) and microwaves (*n* = 3; 2.0%) were less commonly used.

#### 3.2.2 Making bottles one at a time for immediate use

Most parents reported that they either ‘always’ (*n* = 107; 70.9%) or ‘most of the time’ (*n* = 22; 14.6%) prepared bottles one at a time for immediate use. However, a minority (*n* = 22; 14.6%) pre‐prepared bottles half the time or more, 20 (90.9%) of this group used a kettle for the at home experiment. Open text responses noted that having a bottle ready for night‐time feeds was a common rationale (*n* = 11), for example: *‘*We prepare two (bottles) for overnight, sometimes he has one in the night but most of the time they both get used in the morning’ *Mother, age 30, first baby)*. Some parents stated that when multiple bottles were prepared, they were stored in a refrigerator (*n* = 18).

#### 3.2.3 Washing and sterilising all bottle‐feeding equipment

Most parents reported that they washed and sterilised the bottle, teat and bottle parts either most or all of the time (see Table 2). However, the scoop, which is not included in the NHS (2019) advice, was not always routinely washed or sterilised. Levelling equipment, such as a knife, was recorded as ‘not applicable/not answered’ by the majority (*n* = 112, 74.2%), which was explained by some because of using levelling tools being built into PIF packaging. Similarly formula portion pots^1^ and thermal insulated flasks^2^ did not appear to be used by many parents, however most of those who did use them washed them either always or most of the time, but did not routinely sterilise them.

**Table 2:** Washing and sterilising of bottle‐feeding equipment.

		How often do you wash …		How often do you sterilise…	
Equipment	Answer	*n*	%	*n*	%
Bottles	Always/most of the time	147	97.4	142	94.1
	Half the time or less	2	1.3	7	4.6
	N/A or not answered	2	1.3	2	1.3
Teats	Always/most of the time	147	97.3	142	94.1
	Half the time or less	2	1.3	7	4.6
	N/A or not answered	2	1.3	2	1.3
Scoops	Always/most of the time	32	21.2	11	7.3
	Half the time or less	118	78.2	138	91.4
	N/A or not answered	1	0.7	2	1.3
Other parts of the bottle	Always/most of the time	143	94.7	140	92.7
	Half the time or less	5	3.3	8	5.3
	N/A or not answered	3	2	3	2
Knives/levelling implements	Always/most of the time	18	11.9	7	4.7
	Half the time or less	21	13.9	32	21.2
	N/A or not answered	112	74.2	112	74.2
Portion pots	Always/most of the time	67	44.4	24	15.8
	Half the time or less	17	11.3	60	39.7
	N/A or not answered	67	44.4	67	44.4
Flasks	Always/most of the time	46	30.5	19	12.6
	Half the time or less	18	11.9	45	29.8
	N/A or not answered	87	57.6	87	57.7

Open text responses related to *washing* PIF preparation equipment mostly focused on why the scoop was not washed (*n* = 46), which included believing that it was unnecessary (*n* = 28), or a belief that the use of boiling water to prepare PIF made washing unnecessary (*n* = 14): *‘*As the powder goes into hot water it seems unnecessary to wash/sterilise the scoop’ (*Mother, age 37, 2 children)*. Similarly, open text responses relating to not *sterilising* items were focused on the scoop (*n* = 44), formula portion pots (*n* = 20) and thermal insulated flasks (*n* = 11). The belief that sterilising was unnecessary was described again (*n* = 30), as well as the ability of boiling water to sterilise (*n* = 16) and PIF itself not being sterile (*n* = 11), as can be seen in this response: *‘*Formula pot (isn't sterilised) as it gets washed in hot soapy water and then the formula is dispensed into the hot water/bottle. The tin (of PIF) is also not sterile so don't see the point’ *(Mother, age 27, first baby)*.

The most popular method of sterilising was steam (*n* = 86; 57%), followed by cold water solution^3^ (*n* = 52; 34.4%), and microwavable sterilising bags (*n* = 26; 17.2%). Boiling (*n* = 7; 4.6%) and UV sterilisation (*n* = 3; 2%) were less commonly used. A small number of parents (*n* = 7; 4.6%) used ‘self‐sterilising bottles’ which go in the microwave. Most parents who used cold water sterilising solution (36 out of 52; 69.3%) said that they ‘always’ or ‘most of the time’ shake off excess solution from the bottle or teat or rinse them with cool boiled water. However, three people (5.7%) said they only did this half the time or less, and over a quarter (*n* = 13; 25.5%) said that they never did this.

#### 3.2.4 Washing hands and cleaning and disinfecting surfaces

Over three‐quarters of parents (*n* = 118; 78.2%) said that they either ‘always’ or ‘most of the time’ ‘washed their hands before touching any baby feeding equipment/powdered formula’, meaning that over a fifth (*n* = 33; 21.8%) washed their hands half the time or less often. When asked why, a range of practical reasons (*n* = 27) were given, including being in a rush (*n* = 13), forgetting (*n* = 7), trying to comfort an upset baby (*n* = 4) or holding their baby meaning it was impossible to wash their hands (*n* = 3). Other parents noted that there was no need to wash their hands before preparing PIF (*n* = 20), because they had: recently been washed (*n* = 8), used hand sanitiser instead (*n* = 3) or only touched parts of the feeding equipment that the baby's mouth would not make contact with (*n* = 9). Over two thirds (*n* = 105; 69.5%) of parents said that they ‘always’ or ‘most of the time’ cleaned and disinfected surfaces before making their baby's bottle, but over a quarter (*n* = 45; 29.8%) said that they did this only half of the time or less often, including eight (5.3%) who reported that they ‘never’ cleaned and disinfected surfaces before PIF preparation. Discussion in our community analysis group focused on the PIF preparation area of the kitchen being kept clean generally, and not knowing that disinfection was recommended before preparing each bottle.

#### 3.2.5 Barriers and facilitators to following NHS advice

In response to closed questions, the majority of parents reported feeling either ‘very confident’ or ‘quite confident’ in relation to preparing PIF (*n* = 140; 92.7%), and either ‘very’ or ‘quite’ knowledgeable about preparing PIF (*n* = 121; 80.1%). When asked an open question about the benefits of their PIF preparation method, only 12 responses were received. Nine of these reported practices *not* following NHS advice, with one reported following manufacturers' guidance on PIF labelling, and only two actively reporting that they knew and followed NHS advice, for example: *‘*I think my method is long‐winded but I try to do what the guidelines say. A preparation machine would definitely be easier but I haven't used one as I believe the NHS guidelines said not to’. *(Mother, age 33, first baby)*

When asked about barriers for parents preparing formula in line with NHS (2019) advice, the major theme, noted 192 times, was the challenges of following NHS advice in a real‐world setting. This included time pressures (*n* = 70): *‘*Time constraints as a hungry, screaming baby can make you quite anxious to just get them fed’. *(Mother, age 35, two children)*. Alongside this, the challenging context of busy life as a parent was described (*n* = 21): *‘*Managing everything in the day, the demands of parenthood and the sleep deprivation that comes with it’. *(Mother, age 30, first baby)*. Twenty‐one parents stated that the NHS (2019) advice seemed impractical to implement in real world settings (*n* = 21): ‘Too complicated and not manageable in real life. Fine in specific situations (a ward) but much harder in a home with real life set ups/problems/distractions’ *(Mother, age 24, first baby)*.

The second major theme focused on communication of NHS (2019) advice, which was mentioned 131 times. This included lack of clear communication of the advice itself, including a lack of knowledge of the advice and why it was important (*n* = 49). For example, one parent noted:

I didn't know their (sic) were NHS guidelines to be honest. I didn't feel it was treated as a health issue particularly. Very little support from any outside professionals about feeding in general—most support and knowledge gathered from peers. (Mother, age 35, three children)

Other sub‐themes included receiving conflicting advice (*n* = 20), including from different health professionals and between children born several years apart. Finally, the advice was described as being confusing (*n* = 14). The interaction between these overlapping issues was described by one parent:

The NHS guidelines changing a lot doesn't help parents, especially when they have a large age gap. It seems to be one rule for one child and a separate rule for the next one. With my son, bottles could be made up for the entire day and left on the side and warmed up in the microwave. This is no longer considered safe. I also believe that it('s) very difficult to find the actual right information. Midwives will say one thing, doctors will say another and the NHS guidelines say something different, parents don't know which advice to follow. I also believe social media has an impact on how parents make formula, there are a lot of “hacks” for parents to try which make feeding “easier” but do not follow guidelines. Parents who are struggling see these and follow them blindly in a hope it will make things easier for them. (Mother, age 28, two children)

Parents reported that antenatal education did not provide them with sufficient guidance on how to safely prepare PIF, with eight parents reporting a belief that health professionals could not give advice on formula feeding: *‘*(The NHS guidelines are) confusing for one, but the NHS don't promote formula feeding so it's something you have to go out of your way to research—no one tells you how to make it in antenatal classes’. *(Mother, age 29, first baby)*

### 3.3 The at home experiment

#### 3.3.1 Water heating methods and excluded data

Around half of parents used a kettle (*n* = 70; 46.4%), and half a PIF preparation machine (*n* = 75; 49%). Where participants reported temperatures <60°C, research diaries were investigated to ensure that only temperatures that followed the experiment's protocol were included in the analysis. All but one kettle user's temperatures could be easily explained, for example lower temperatures relating to a delay of more than 30 min in boil to pour time. The kettle user with a temperature below 40°C reported that she thought the thermometer was faulty, so this reading was excluded from analysis. Eleven PIF preparation machine temperatures were investigated, including three participants who reported water temperatures below 40°C. Two formula machine users misunderstood the experiment protocol and took the temperature after adding cold water to the ‘hot shot’, these participants were asked to repeat the experiment and the new readings were included in the analysis instead. A third machine user explained that she routinely dispensed a ‘hot shot’ and then added cool water *before* adding PIF as the manufacturer's guidance for the PIF she uses required water to be cooler. This result was also excluded from the experiment analyses. Eight machine users reporting temperatures between 40°C and 60°C (104–140 °F) reported that they had followed the experimental protocol, and were invited to repeat the experiment. Four did so and three continued to have readings below 70°C (58.5°C, 58.8°C and 60.3°C), while one reported an initial temperature of 59.9°C, but a second temperature of 72.7°C, suggesting potential wide variation in temperatures. However, the first temperature results were used in the analysis, as these four users *had* followed the experiment protocol. The final sample size for the experiment was 143 (*n* = 69 kettle; *n* = 74 PIF preparation machine). Due to small group numbers, baby kettle (*n* = 2; 2.4%) and instant boiling tap (*n* = 1; 0.7%) users were excluded from the group comparison analyses. Three people did not answer the question.

#### 3.3.2 Water temperature

The mean water temperature reported in the experiment was 70.4°C (SD:11.1, range: 40.1–99.5). The mean temperature reported by the kettle users was >9°C higher than the formula preparation machines (see Table 3). A t‐test with a 95% confidence interval showed a significant difference was found between the groups [*t*(141) = 5.64, *p* < 0.001]. Further, the Cohen's effect size value was 0.94, suggesting a meaningful significance between the two groups.

**Table 3:** Experiment temperatures in degrees Celsius for different heating methods.

Heating method	*n*	*M*	SD	Range
Kettle Users	69	75.29	12.88	40.1–93.2
Formula preparation machines (‘hot shot’ only)	74	65.78	6.39	50.1–99.5

Data were grouped into those who reported a temperature of ≥70°C, and those who reported a lower temperature. 78.3% (*n* = 54) of kettle users reported temperatures ≥70°C, compared with only 14.9% (*n* = 11) of formula machine users. A *χ* ^2^ test showed that this difference was statistically significant [*χ* ^2^(1), *N* = 143 = 57.8, *p* < 0.001]. The boil to pour time of kettle users that reported temperatures <70°C were examined (*n* = 14 of 15 parents). The mean boil to pour time of the <70°C group was 34 min (SD:39.7, range 3–157 min) compared to 8 min for the ≥70°C group (SD:8.16, range 0–33 min). Eight parents reported boil to pour times ≤30 min and temperatures <70°C, with four in the 60–70°C range (68.8°C, 21 min; 69.9°C, 30 min; 66.7°C; 30 min, 61.8°C, 30 min). Research diaries revealed that four used a mixture of hot and cool boiled water (*n* = 4), and one decanted the water into a bottle and used an insulated bottle warmer after boiling.

In the kettle group, differences in temperature were explored based on the volume of water heated in the kettle and the volume of water poured into the bottles (data available for 67 participants). Mean temperature was greater where larger volumes of water were boiled (>1 L *n* = 15 *M*:78, SD: 7.62; 1 L exactly *n* = 33 *M*:75.67, SD: 13.2; <1 L *n* = 19 *M*:71.75, SD: 15.64), however an ANOVA found that this difference was not statistically significant, [*F*(2, 64) 1.01, *p* = 0.36]. Similarly, a *χ* ^2^ test did not find a significant relationship between volume of water heated and likelihood of achieving a temperature ≥70°C [*χ* ^2^(2), *N* = 67 = 1.19, *p* = 0.52]. However, an ANOVA was performed to explore the relationship between volume of water heated (>1 L, 1 L exactly and <1 L) and resultant temperature, controlling for boil‐to‐pour time and found a significant difference between the three groups [*F*(3, 60) 3.15, *p* = 0.03]. Furthermore, a Pearson's correlation showed a positive correlation between the volume of water poured into the bottles and temperature recorded (greater volumes were associated with greater temperatures), but this relationship was not statistically significant [*r*(68) = 0.69, *p* = 0.57].

## 4 DISCUSSION

Half of parents in our study used a PIF preparation machine, which is not recommended as part of NHS (2019) safer feeding advice, regardless of the water temperature produced by preparation machines. We identified a statistically significant finding that, in real world conditions using 74 unique PIF preparation machines, it was rare (<15%) for a minimum temperature of 70°C to be recorded at the time when parents would typically add the PIF. This has been identified in previous laboratory‐based research, which suggested the small volume of water in a ‘hot shot’ will cool to around 60°C within the 2 min that manufacturers recommend PIF is added (Crawley et al., 2022). Accordingly, these machines may not kill all bacteria that are present in PIF. This is concerning as research indicates around half of parents in the UK use a PIF preparation machine (Brown et al., 2020). Furthermore, recent research has recommended that PIF preparation advice should be amended to recommend the use of a temperature of >85°C, because reconstituted PIF made with water at 70°C cools to 57.5–60.0°C within 2 min, potentially failing to kill all bacteria present (Losio et al., 2018). The only nutrient in the PIF likely to be significantly impacted is vitamin C, which is unlikely to be reduced below recommended levels during PIF reconstitution (WHO, 2006).

In addition to bacteria which are contained within PIF, additional bacterial contamination can occur through inadequate washing and sterilising of PIF preparation equipment (Crawley et al., 2022), unclean hands (Cho et al., 2019), preparing PIF in advance and contaminated work surfaces (WHO, 2007). Together it is estimated that bacterial contamination of PIF results in an additional 3000 hospital admissions and 10,000 General Practitioner visits each year in the United Kingdom due to gastrointestinal infections (Renfrew et al., 2012), no doubt causing significant distress to parents and carers. Barriers to safer PIF preparation identified in our study centred on parents not knowing or understanding what safer PIF preparation guidance was, and being unfamiliar with the risks of bacterial contamination, combined with the time pressures of caring for a baby. This failure to adequately communicate risk resulted in 30% making PIF in advance, and many not always washing and sterilising all equipment. This was a particular issue in relation to scoops, which are not currently included in the NHS (2019) advice, with 86% never sterilising, despite evidence that this can transmit bacteria to PIF (Cho et al., 2019). If parents were aware of the potential for their baby to become seriously unwell as a result of PIF bacterial contamination, they would be more likely to prioritise following safer PIF guidance (Michie et al., 2014). Accordingly, there is an urgent need for guidance on safer PIF preparation and information on the risks of not doing so to be transmitted to parents, including through sufficient clear statements on PIF labelling and via health professionals.

### 4.1 Strengths and limitations

This is the first study we are aware of to use community scientists to collect food safety data relating to PIF preparation in the home, providing data showing real world evidence from 74 unique PIF preparation machines, which show potential for a direct impact on the incidence of food borne illness. Furthermore, PIF users were involved in the development of our data collection tools, resulting in enhanced clarity, although we did not collect data on whether participants were members of the study's closed Facebook group or not to be able to assess any potential bias introduced. It is sometimes suggested that community science approaches allow the introduction of biases, including social desirability bias. However, the large number of respondents who reported practices outside of NHS (2019) safer PIF preparation guidance suggests that social desirability bias was not a significant issue in our study. We also received a high response rate, with three quarters of those requesting a study pack completing data collection, allowing us to identify statistically significant results. Finally, five community scientists were involved in the analysis and interpretation of open text responses, increasing the validity of our findings and four were involved in developing this article.

Limitations include the use of basic digital home food thermometers, which were not scientific/medical grade, and were not calibrated after purchase. One kettle user reported a faulty thermometer and was excluded from analysis and two PIF preparation machine users reported that their thermometers took up to 15 s to stabilise at a temperature. This may have affected their results, although the manufacturer recommended adding PIF to the ‘hot shot’ within 2 min at the time of study design. The manufacturer has subsequently changed their advice, recommending that PIF is added to the bottle before water, although this is not considered best practice (NHS, 2019), and thus it would not be ethical to do research on the impact of this new instruction. While our design involved collecting data on the temperature of water used to reconstitute PIF, and the extent that it met the minimum recommended temperature of 70°C, we did not collect data on the microbiological effects of the lower than optimal water temperatures recorded or any subsequent food borne illness. Given the established scientific evidence which has informed recommended water temperatures, it is our opinion that this data is not necessary to prove the risks of poor compliance with WHO (2007) and NHS (2019) advice.

While we compared the temperatures obtained by kettle users and PIF preparation machine, PIF machine users were asked to undertake the experiment *separately* to preparing a bottle, while kettle users were asked to do so *simultaneously*, potentially resulting in kettle users experiencing more stress, such as from preparing a bottle of PIF for a hungry baby. Despite this, significant differences were still found. However, to allow for a more direct comparison, future research should use infrared (scientific/medical grade) thermometers, which could measure the temperature of water in the actual bottle of PIF being prepared for feeding. Parents reported increased risky PIF preparation practices overnight, but were only asked to provide temperature data at one point in time in this study, due to the limited incentives available. These low value (£5) incentives and convenience sample using the study team's social media networks are also likely to have enhanced self‐selection bias, resulting in our sample containing a high proportion of participants who were highly educated and older, which has been associated with increased knowledge of PIF preparation (Calamusa et al., 2009). Social desirability bias may also have resulted in under reporting of risky PIF preparation practices. Finally, an error in our survey tool meant that ethnicity data was not clear enough to report.

## 5 CONCLUSION

Parents should be advised that many PIF preparation machines will not produce water that meets the minimum temperature needed to kill any bacteria present in PIF, which is not and cannot be made to be sterile, and instead should be advised to follow NHS (2019) advice, including heating water in a kettle so that it is >70°C, and, per Losio et al. (2018), ideally >85°C. There is an urgent need for stronger consumer protections with respect to the marketing of PIF and PIF preparation devices to further protect infants from PIF‐related bacterial contamination, which can result in serious ill health and even death. This should ensure that PIF labelling is compliant with the WHO (1981) guidance (WHO Europe, 2022). Accordingly, countries that have not yet legislated to ratify the WHO (1981) code, including the UK, should do so as a matter of urgency. We also recommend that PIF labelling should very clearly state the importance of hand washing, to reduce the potential for contamination of scoops and PIF. To mitigate an additional route of bacterial contamination, the routine sterilising of scoops should be added to PIF preparation guidance. These important public health messages should also be transmitted through antenatal and post natal infant feeding support, which should be facilitated through full and robust implementation of the UNICEF Baby Friendly Initiative. Finally, bacterial gastrointestinal infections in infants, particularly those resulting in hospitalisation, should be mapped to PIF and PIF preparation equipment to provide information that could further improve the safety of PIF preparation.

## AUTHOR CONTRIBUTIONS

Aimee Grant contributed to conceptualization, data curation, formal analysis, funding acquisition, investigation, methodology, supervision, writing original draft and writing review and editing. Sara Jones contributed to conceptualization, data curation, formal analysis, funding acquisition, investigation, methodology, writing original draft and writing review and editing. Vicky Sibson contributed to conceptualization, data curation, funding acquisition and writing review and editing. Rebecca Ellis contributed to data curation, formal analysis and writing review and editing. Abbie Dolling contributed to formal analysis and writing review and editing. Tara McNamara contributed to formal analysis and writing review and editing. Jonie Cooper contributed to formal analysis and writing review and editing. Susan Dvorak contributed to formal analysis and writing review and editing. Sharon Breward contributed to conceptualization, data curation, funding acquisition and writing review and editing. Phyll Buchanan contributed to conceptualization, data curation, funding acquisition and writing review and editing. Emma Yhnell contributed to conceptualization, data curation, funding acquisition and writing review and editing. Amy Brown contributed to conceptualization, data curation, funding acquisition, supervision and writing review and editing.

## CONFLICT OF INTEREST STATEMENT

The authors declare no conflict of interest.

## ETHICS STATEMENT

The research received ethical approval from Swansea University School of Health and Social Care Research Ethics Committee.

## Supporting information

Supporting information.

Supporting information.

## Data Availability

The data that support the findings will not be made available.

## Footnotes

1 Formula portion pots are small plastic tubs into which carers can decant individual portions of PIF. They are commonly used when using PIF outside of the home. They are not recommended by the NHS (2019).

2 Thermal insulated flasks may be used by those preparing PIF to attempt to keep water above 70°C when preparing PIF without access to a kettle.

3 Cold water solution is a way of sterilising equipment, including that used for infant feeding, using a mixture of cold water and a sterilising agent, which can either be in liquid or tablet form. The most common sterilising agent in the United Kingdom, Milton, contains sodium hypochlorite and sodium chloride.
