# Supplementary material for: The safety of at home powdered infant formula preparation: A community science project
Source: Matern Child Nutr. 2023 Oct 4;20(1):e13567. doi: 10.1111/mcn.13567 (PMC10750023; doi:10.1111/mcn.13567)
Supplement: Supplementary file 1 — Supporting information. [file MCN-20-e13567-s001.pdf]

FINDING THE FORMULA STUDY: INSTRUCTION SHEET

USE THIS SHEET IF YOU USE A KETTLE /BABY KETTLE/HOT WATER TAP OR SIMILAR

In this experiment, we will ask you to:

- Make up a bottle of formula like you would usually (we'll call this the 'formula bottle'. At the same time, or as soon as possible afterwards, use a second bottle to take the temperature of the water you used to prepare the formula (we'll call this the 'test bottle')
- Write down some details to add to the research diary when you complete it later

The reason we ask you to use a test bottle is because the thermometer could introduce bacteria which could make your baby sick.

The thermometer should never go into the formula bottle.

Step One

Gather all the supplies you need to make up a bottle of formula, and an extra empty bottle.

You'll have one bottle (which we'll call the 'formula bottle') and another one beside it (which will be the 'test bottle').

You will also need:
- a pen or your phone
- the food thermometer that we sent you in the post. (see instructions below)

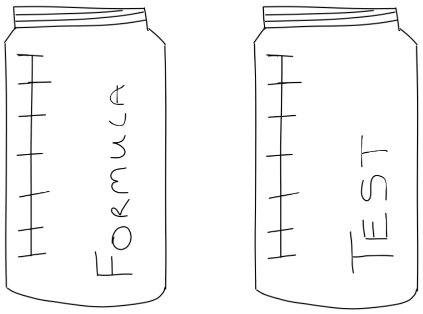

Step Two

Heat the water as you would normally.

Please take care when handling hot water.

Turn the thermometer on. And ensure it is set to C

Step Three

Fill both the formula bottle and the second test bottle with the same amount of water.

For example, if you want to make 7oz of formula, pour 7oz of water into both bottles

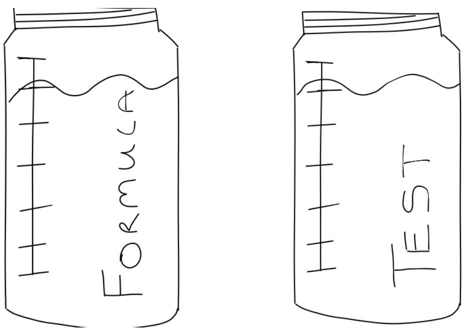

How to use the thermometer:

1. Turn the thermometer on using the on/off button.

2. Ensure it is set to C (it shows it on the bottom right-hand corner of the screen). This can be changed by pressing the C/F button.

3. Place the thermometer into the test bottle

4. Wait for 15 seconds for the reading to steady then write it down here or on your phone.

Step Four

Add formula to the formula bottle only then **immediately** take the temperature of the water in the test bottle

(note: there is no formula in the test bottle - only water)

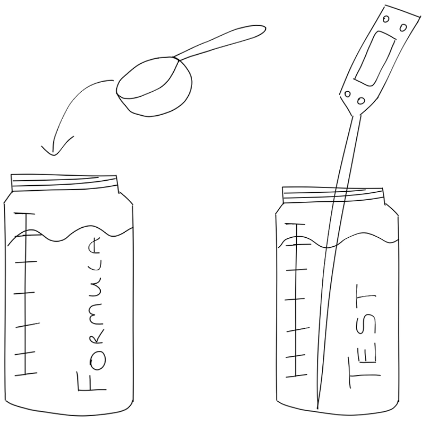

Step Five

Write down some details to add to the research diary when you complete it later:

**What time did you boil the kettle? (If you used a kettle)** Include if it is am or pm	
**Volume of water heated** (eg. 1 litre). If you use a machine or you're not sure you can leave this blank	
**What time did you pour the water into the bottles?**	
**Volume of water in bottle** (eg: 4oz)	
**Temperature selected on baby kettle.** Leave this blank if this doesn't apply to you	
**Temperature recorded on the thermometer** (eg: 72 degrees)	

Step Six

Discard the water in the test bottle and clean/sterilise the bottle

After the experiment

As soon as you can, please complete your online research diary using the link/QR code; this link has also been emailed to you from Sara Jones (s.w.jones@swansea.ac.uk). It should take no more than 30 minutes to complete.

QUESTIONS?

Email Sara (s.w.jones@swansea.ac.uk) or
Aimee (Aimee.Grant@swansea.ac.uk)
or FB message us @findingtheformula-community science group
