# Supplementary material for: The safety of at home powdered infant formula preparation: A community science project
Source: Matern Child Nutr. 2023 Oct 4;20(1):e13567. doi: 10.1111/mcn.13567 (PMC10750023; doi:10.1111/mcn.13567)
Supplement: Supplementary file 2 — Supporting information. [file MCN-20-e13567-s002.pdf]

**FINDING THE FORMULA STUDY: INSTRUCTION SHEET**

**USE THIS SHEET IF YOU USE A FORMULA PREPARATION MACHINE THAT DISPENSES WATER ONLY**

**(You cannot do the experiment if the machine has formula inside or dispenses formula e.g. Baby Brezza)**

In this experiment, we will ask you to:

- Use an empty bottle to take the temperature of the water your preparation machine produces (we'll call this the 'test bottle')
- Write down some details to add to the research diary when you complete it later

The reason we ask you to use a test bottle is because the thermometer could introduce bacteria which could make your baby sick.

The thermometer should never go into a bottle you use to make formula.

## Step One

You will need an empty bottle that will not be used to make a bottle of formula.

You'll have one bottle (which will be the 'test bottle').

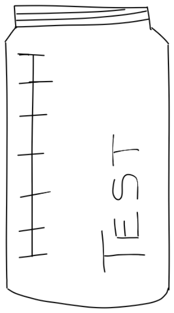


You will also need:
- a pen or your phone
- the food thermometer that we sent you in the post. (see instructions below)

## Step Two

Keep the settings the same on your fomrula preparation machine as the last bottle.

**Please take care when handling hot water.**

Turn the thermometer on. And ensure it is set to C

## Step Three

Add the hot shot only to the empty bottle. No formula.

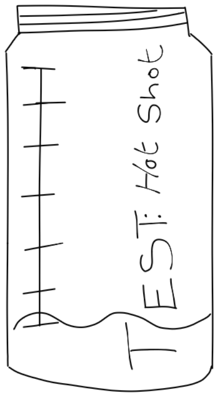


## How to use the thermometer:

| |
|---|
| 1. Turn the thermometer on using the on/off button. |
| 2. Ensure it is set to C (it shows it on the bottom right-hand corner of the screen). This can be changed by pressing the C/F button. |
| 3. Place the thermometer into the test bottle |
| 4. Wait for 15 seconds for the reading to steady then write it down here or on your phone. |

**Please turn to page 2**

## Step Four

**Immediately** take the temperature of the water in the test bottle.

(note: there is no formula in the test bottle - only water)

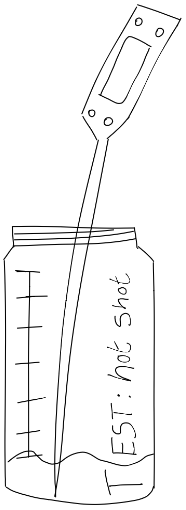


## Step Five

Write down some details to add to the research diary when you complete it later:

| | |
|---|---|
| **Time:** Include if it is am or pm | |
| **Volume of water in bottle (e.g. 4oz)** If you're not sure just write 'hot-shot' | |
| **Temperature selected on baby machine.** Leave this blank if this doesn't apply to you | |
| **Temperature recorded on the thermometer** (eg: 72 degrees) | |

## Step Six

Discard the water in the test bottle and clean/sterilise the bottle

## After the experiment

As soon as you can, please complete your online research diary using the link/QR code; this link has also been emailed to you from Sara Jones (s.w.jones@swansea.ac.uk). It should take no more than 30 minutes to complete.

## QUESTIONS?

Email Sara (s.w.jones@swansea.ac.uk) or
Aimee (Aimee.Grant@swansea.ac.uk)
or FB message us @findingtheformula-community science group
